# Acupoint injection therapy for diabetic retinopathy: A protocol for systematic review and meta-analysis

**DOI:** 10.1097/MD.0000000000024119

**Published:** 2021-01-08

**Authors:** Yanni Zhou, Hui Li, Lisi Luo, Yue Chen, Qiang Chen, Wei Bian, Yanlin Yang, Ju Tang

**Affiliations:** a Chengdu University of Traditional Chinese Medicine, Chengdu, Sichuan Province; b Zhongshan Ophthalmic Center, Sun Yat-sen University, Guangzhou; c Guangzhou First People's Hospital, Guangzhou, Guangdong Province; d Chongqing Yongchuan Traditional Chinese Medicine Hospital, Chongqing, China.

**Keywords:** acupoint injection, diabetic retinopathy, protocol, systematic review

## Abstract

**Background:**

Diabetic retinopathy (DR) is a common diabetic microvascular complication, and it is also the main cause of blindness in adults. At present, some studies have reported acupoint injection for the treatment of DR. However, the effectiveness and safety are still uncertain. This study aims to evaluate the efficacy and safety of acupoint injection for the treatment of patients with DR.

**Methods:**

The databases of English databases (PubMed, Embase, Cochrane Library, Web of Science) and Chinese databases (China National Knowledge Infrastructure, Chinese Scientific Journal Database, Wanfang Database, and Chinese Biomedical Literatures Database) will be retrieved. Published randomized controlled trials and quasi-randomized controlled trials on the topic will be retrieved by 2 investigators independently. We will apply a fixed-effect model or random effect model basis on the heterogeneity test and employ with RevMan 5.3 software for data synthesis. The total effective rate will be selected as the primary outcome, visual acuity, hemorrhage areas, exudates, capillary nonperfusion areas, hemorheological indicators, mean defect of visual field, glycated hemoglobin, and adverse events as secondary outcomes.

**Results:**

This study will comprehensively summarize the high-quality trials to determine the effectiveness and safety of acupoint injection treatment for patients with DR.

**Conclusion:**

The systematic review of this study will summarize the currently published evidence of acupoint injection treatment for DR to further guide its promotion and application.

**Protocol registration number:**

INPLASY2020110026

## 1 Introduction

Diabetes mellitus (DM) is a group of chronic metabolic diseases caused by genetic, environmental, and autoimmune diseases. The dramatic increase in the incidence of DM is becoming a major public health issue. Parallel with the growing DM pandemic, the occurrence of diabetic retinopathy (DR) is also increasing.^[1]^ DR is an eye disease caused by impaired glucose metabolism in diabetic patients. Hemorheological changes in diabetic patients include increased blood viscosity and increased platelet aggregation, which will lead to retinal hemorrhage, edema, exudation, and microangioma. The comprehensive results of DR are capillary occlusion, retinal tissue hypoxia, and the hypoxic tissue produces neovascular factors to stimulate the growth of retinal neovascularization, neovascularization is prone to rupture and hemorrhage, which eventually leads to vitreous hemorrhage and traction retinal detachment.^[2–4]^ If there is no timely treatment, it will often lead to vision loss and even blindness.^[5]^ DR is the most common microvascular complication of diabetes.^[6]^ During the first 2 decades of disease, nearly all patients with type 1 diabetes and 60% of patients with type 2 diabetes have retinopathy.^[7]^ According to epidemiological statistics, the global prevalence rate of DR is 34.6%, and in developed countries, the prevalence rate of DR is close to 40.3%.^[8]^ According to the survey, the cost of diabetic vascular complications accounts for 80% of the direct medical expenses, causing a huge economic burden to the society.^[9]^ Therefore, early prevention and treatment are necessary. However, conventional treatment options are limited. At present, laser therapy and antivascular endothelial growth factor therapy are the mainstream therapies for DR, but they can cause other complications.^[10–12]^

In recent years, acupoint injection has been gradually used in the clinical treatment of DR, and has achieved good results in delaying the progression of DR and restoring vision.^[13,14]^ Acupoint injection is a complementary alternative therapy based on the combination of acupoints, drugs, and meridians,^[15]^ by injecting a small amount of liquid drugs into specific acupoints and generating stimulation to treat diseases.^[16]^ Modern theoretical research shows that acupuncture at acupoints around the eyes can dredge local Qi and blood, and improve blood circulation in the retina.^[13]^ The topical application of the drug to the eye may promote blood microcirculation, improve vascular endothelial function, and protect the blood-retinal barrier. Injecting drugs into acupoints around the eyes has the dual effects of acupuncture and drugs. The combination of the 2 can make the drug go directly to the disease site along the meridians, and the effect can be achieved in a few minutes. Moreover, the drug is retained in the meridians, so that the acupuncture sensation should be exerted and continued, so as to stimulate the meridian qi and strengthen the curative effect, and achieve the purpose of treating DR.^[17]^

Some clinical trials of acupoint injection therapy for DR have been reported; however, a systematic evaluation of the efficacy of acupoint injection therapy for DR remains to be conducted. Therefore, this study will evaluate the effectiveness and safety of acupoint injection therapy for DR according to the authoritative Cochrane recommendations. This information will provide an important reference for clinical decision-making.

## 2 Methods

### 2.1 Study registration and ethics

The protocol was registered on the International Platform of Registered Systematic Review and Meta-analysis Protocols (registration number: INPLASY2020110026; https://inplasy.com.). The data of our study will be obtained from published literature, so ethical approval will be not required.

### 2.2 Type of studies

#### 2.2.1 Inclusion criteria

All relevant randomized controlled trials and quasi-randomized controlled trials will be included. The language of the trials to be included only Chinese or English. Without any date of dissemination or restriction of publication type.

#### 2.2.2 Exclusion criteria

Following studies will be excluded:

1. Repeated publications;
2. Review of literature and cases;
3. Incomplete literature;
4. Nonrandomized controlled trials;
5. Patients combined with other basic diseases.

### 2.3 Type of participants

We will include patients with a diagnosis of DR. The diagnostic criteria of DR in the selected literature should meet the internationally recognized standards. There will be no restrictions on the length of treatment and duration of follow-up.

### 2.4 Interventions and controls

Interventions included treatment with acupoint injection. The drug types for acupoint injection, the acupuncture points for injection, treatment frequency, and duration of treatment will not be restricted. The use of acupoint injection is the only difference between intervention and control. The control interventions will include: positive interventions, placebo, no intervention. The choice of specific forms are as follows:

1. acupoint injection vs positive interventions;
2. acupoint injection + positive interventions vs positive interventions;
3. acupoint injection vs placebo;
4. acupoint injection vs no intervention.

### 2.5 Types of outcome measures

#### 2.5.1 Primary outcomes

The primary outcomes were the total effective rate. The total effective rate was defined as the number of patients who showed improvement in retinal vascular-related abnormalities.

#### 2.5.2 Secondary outcomes

1. visual acuity;
2. retinal vascular abnormalities: hemorrhage areas, exudates, capillary nonperfusion areas, hemorheological indicators;
3. mean defect of visual field;
4. glycated hemoglobin;
5. adverse events.

### 2.6 Search strategy

#### 2.6.1 Electronic searches

Two investigators (YNZ and HL) will execute the structured and systemic literature retrieval without interfering with each other in the following electronic bibliographic databases until November 2020.: PubMed, Embase, the Cochrane Library, Web of Science, China National Knowledge Infrastructure (CNKI), Chinese Scientific Journal Database (VIP), Wanfang Database, and Chinese Biomedical Literatures Database (CBM). The search period of our study will be from the establishment of the database to November 2020. The search strategy that will be run in the PubMed and tailored to the other database when necessary is presented in Table 1.

**Table 1:** Initial draft of the search strategy with PubMed as an example.

Number	Search items
#1	Diabetic Retinopathy[Title/Abstract]
#2	Diabetic Retinopathies[Title/Abstract]
#3	Diabetic eye disease[Title/Abstract]
#4	#1 OR #2 OR #3
#5	Acupoint injection [Title/Abstract]
#6	Point injection [Title/Abstract]
#7	Acupuncture point injection [Title/Abstract]
#8	#5 OR #6 OR #7
#9	Randomized controlled trial[Title/Abstract]
#10	Controlled clinical trial [Title/Abstract]
#11	Randomized [Title/Abstract]
#12	Randomly [Title/Abstract]
#13	Clinical Trials [Title/Abstract]
#14	#9 OR #10 OR #11 OR #12 OR #13
#15	#4 AND #8 AND #14

#### 2.6.2 Searching other resources

Relevant documents will also be retrieved, such as medical textbooks, clinical handbooks. relevant conference proceedings, references list of eligible studies, and dissertations of degrees. Meanwhile, experts in relevant fields will be contacted to obtain important information that cannot be found from the retrieval.

### 2.7 Data collection and analysis

#### 2.7.1 Selection of studies

**Figure 1:**
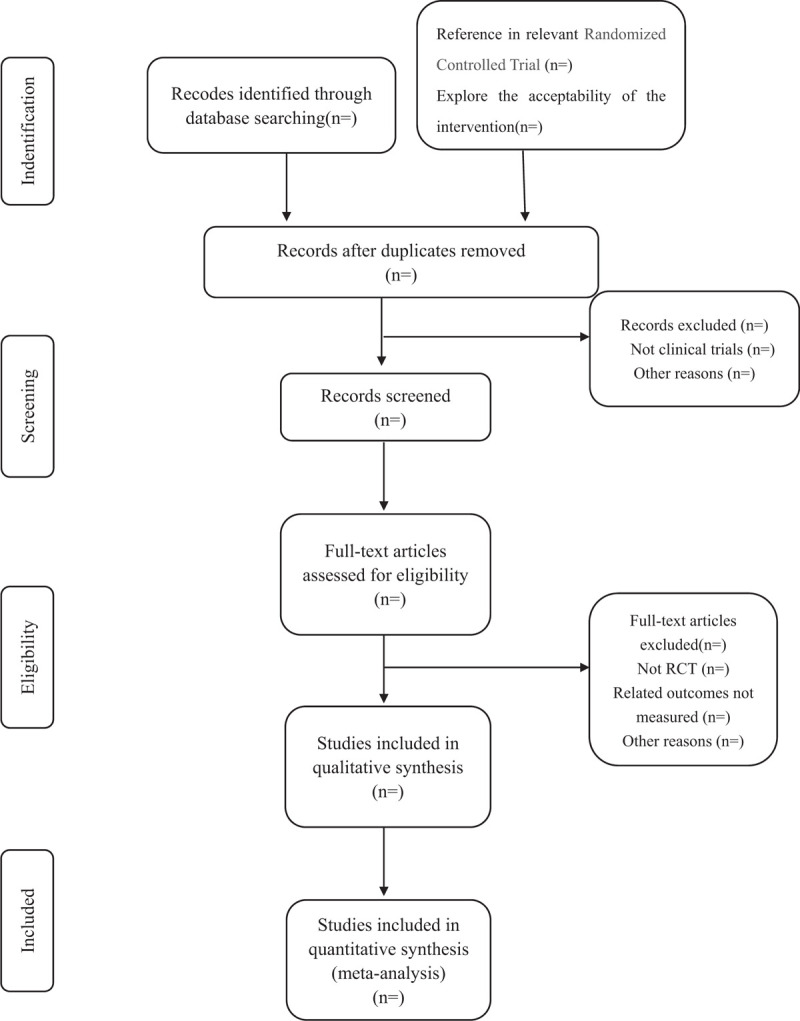
Study selection flow chart.

The researchers will import all the searched references into Endnote X8 software for management, and delete the duplicate contents. Two reviewers (YNZ and HL) will independently review the titles, abstracts, and keywords of the literature based on inclusion and exclusion criteria to exclude irrelevant studies. Excluded explanations will be recorded in the excel data set. In addition, the full text of articles should be preserved and archived for further investigation. Any difference between the 2 researchers during the research process will be decided by the other author (LSL). Figure 1 displayed the flow diagram of our study in detail.

#### 2.7.2 Data extraction and management

Two researchers (YNZ and HL) will read all the selected study, and independently extract the following information: general information (title, author, year of publication, journal); study details (design, randomized method); characteristics of participants (age, gender, sample size, course of disease, inclusion/exclusion criteria); intervention measures (acupoints, single treatment time, course of treatment, combination therapy, follow-up time points); outcome (primary and secondary outcomes). If researchers cannot reach an agreement, a third researcher (LSL) will make the final decision. If the reported data is insufficient or ambiguous, we will contact the original study author for more information.

#### 2.7.3 Risk of bias assessment

Two independent reviewers (YC and QC) will use the Cochrane “bias risk assessment” tool to assess the bias risk of the selected trials. The tool assesses the risk of bias mainly in the following 7 aspects: random sequence generation, allocation concealment, the blinding method for patients, researchers and outcomes assessors, incomplete result data, and selective reports. As recommended by the Cochrane manual, the risk of bias in each of these areas will be assessed as low or high depending on whether the criteria were met or not met, and the lack of information will be recorded as unclear. Differences will be resolved by discussion with the third author until consensus be achieved.

#### 2.7.4 Data analysis and synthesis

We will use RevMan5.3 software for meta-analysis. For dichotomous data (eg, effective and ineffective), we will calculate risk ratio and 95% confidence intervals (CIs). For continuous data, when the measurement method and unit are consistent, we will calculate the weighted mean difference and 95% CIs. When the measurement methods and units are inconsistent or the mean values of different experiments differ greatly, we will use the standardized mean difference and 95% CIs as the composite statistics.

#### 2.7.5 Investigation of heterogeneity

Statistical heterogeneity will be assessed by *χ*^2^ and *I*^2^ statistical tests. Where *P* value ≥.1 and *I*^2^ ≤ 50%, there is no obvious statistical heterogeneity among the studies. On the contrary, where *P* value <.1 or *I*^2^ > 50% indicates a considerable heterogeneity. Meta-analysis will be performed when the statistical heterogeneity is acceptable (*P* value ≥.1 and *I*^2^ ≤ 50%), otherwise, subgroup analysis will be applied to explore the influence of potential factors on the outcome measures. We will conduct sensitivity analyses by omitting studies one by one to probe the impact of an individual study. If a meta-analysis cannot be performed, we will conduct descriptive analysis instead.

#### 2.7.6 Subgroup analysis

To resolve some potential problems, we will perform a subgroup analysis. First, we will compare the results of different drug injections at acupoints. Second, we will compare the results of acupoint injection alone with those combined with other active treatments.

#### 2.7.7 Sensitivity analysis

A sensitivity analysis will be performed to test the robustness of the review result and to detect the source of heterogeneity. This can be done by excluding trials with a high risk of bias or eliminating each study individually. And, the impact of methodological quality, sample size, and missing data will be assessed. Then the analysis will be repeated after the exclusion of low methodological quality studies and the results compared with the previous meta-analysis.

#### 2.7.8 Reporting bias assessment

When we select >10 studies consistent with conditions, a funnel plot and Egger regression test will be performed to appraise the reporting biases.

#### 2.7.9 Evidence quality

The quality of evidence will be ranked as 4 levels: high, moderate, low, and very low according to the Grading of Recommendations Assessment, Development and Evaluation (Version 3.6, The grading of recommendations assessment, development, and evaluation Working Group) instrument for included studies. It is based on 5 key domains: risk of bias, consistency, directness, precision, and publication bias.

## 3 Discussion

DR is a kind of blinding eye disease that can be prevented and controlled. DR is a common cause of vision loss in adults, and the prevalence has been increasing since 1990. In particular, the number of blindness caused by DR increased significantly.^[18]^ Acupoint injection therapy is based on the basic theory of acupuncture, and under the guidance of meridian theory, it combines meridians, acupoints, and drug, which will amplify the stimulation effect of acupuncture and drugs, so as to improve the pathological condition and regulate the body.^[19]^ This therapy has the advantages of short course of treatment, good therapeutic effect, low cost, and good safety. It has been widely used in the clinical treatment of various diseases. Research showed that the therapeutic effect of Acupoint Injection on DR was significantly better than that of conventional drug treatment. After treatment, the retinal circulation time was significantly shortened, the macular thickness and macular edema were significantly improved, which was worthy of clinical application.^[20]^ The mechanism may be to stimulate the nerve endings of the superficial temporal artery by stimulating the temporal, thereby regulating the choroidal autonomic nerves, improve the vasomotor function, and finally reduce the probability of retinal microcirculation abnormalities, promote retinal rehabilitation.^[21,22]^

At present, many randomized controlled trials have shown the efficacy and safety of acupoint injection in the treatment of DR, but there is no relevant systematic evaluation. This is the first systematic evaluation and meta-analysis to provide reliable evidence for clinical promotion of acupoint injection therapy for DR. As we are not good at other languages, the literature we search for is limited to Chinese and English, which will cause some prejudice. Besides, the limitation of the sample size also leads to the instability of the reliability of the conclusion.

## Author contributions

**Conceptualization:** Yanni Zhou.

**Data curation:** Yanni Zhou, Hui Li, Lisi Luo.

**Formal analysis:** Yanni Zhou, Hui Li, Yanlin Yang, Ju Tang.

**Funding acquisition:** Yanni Zhou.

**Methodology:** Yanni Zhou, Hui Li, Lisi Luo, Yanlin Yang, Ju Tang.

**Project administration:** Yanni Zhou, Hui Li, Wei Bian.

**Software:** Yue Chen, Qiang Chen.

**Supervision:** Wei Bian.

**Validation:** Yanni Zhou, Hui Li.

**Writing – original draft:** Yanni Zhou.

**Writing – review & editing:** Wei Bian.
